# Cultural Adaptation and Reliability Testing of the Coeliac Disease Food Attitudes and Behaviours Scale in Brazil

**DOI:** 10.3390/nu18010162

**Published:** 2026-01-03

**Authors:** Camila dos Santos Ribeiro, Eduardo Yoshio Nakano, Renata Puppin Zandonadi

**Affiliations:** 1 Department of Nutrition, University Center of the Institute of Higher Education of Brasília, Brasília 70200-730, Brazil; 2 Department of Statistics, University of Brasília, Brasília 70910-900, Brazil; nakano@unb.br; 3 Department of Nutrition, University of Brasília, Brasília 70910-900, Brazil

**Keywords:** coeliac disease, CD-FAB, questionnaire, gluten-free diet, eating behaviours

## Abstract

**Background**: Coeliac disease (CD) is an autoimmune chronic condition that requires a strict diet without gluten as a single effective treatment. However, adherence to a life-long gluten-free diet (GFD) may promote psychological suffering and disordered eating. The Coeliac Disease Food Attitudes and Behaviours Scale (CD-FAB) is the only instrument specifically designed to evaluate these aspects in individuals with CD. **Objective:** This study aimed to translate the CD-FAB and test its reliability in the Brazilian population. **Methods**: The research was conducted in three stages: (i) translation and cultural adaptation of CD-FAB into Brazilian Portuguese; (ii) pretesting with Brazilian individuals diagnosed with CD to assess item understanding; and (iii) psychometric evaluation through reproducibility (test–retest reliability) via intraclass correlation coefficient (ICC) and internal consistency via Cronbach’s alpha. **Results**: The CD-FAB was successfully adapted to the Brazilian CD population (Child CD-FAB-BR) and demonstrated strong internal consistency (α = 0.807) and an intraclass correlation coefficient (ICC = 0.928). **Conclusions**: The Brazilian version of CD-FAB proved to be reliable for assessing food attitudes and behaviours in people with CD, potentially allowing for the identification of factors to guide research and clinical practice by driving behaviour-based strategies to improve nutritional education and strategic policies for coeliac patients. Studies with larger and more diverse samples are recommended to provide external validation of the instrument.

## 1. Introduction

Adhering to a gluten-free diet (GFD) is crucial for treating coeliac disease, a chronic autoimmune condition related to gluten ingestion in individuals with a genetic predisposition [1,2]. It is estimated that CD affects up to 1.4% of the global population, but it is considered a neglected and underdiagnosed condition [1,2]. To date, only a strict and permanent GFD is capable of reversing intestinal mucosal damage and symptoms, which can manifest as intestinal or extraintestinal [1,3,4]. GFD adherence poses a challenge encompassing both individual and collective aspects, including education, self-perception, economic and physical access to gluten-free products, access to qualified professionals, accidental gluten consumption, inadequate food labelling, and other factors [5,6]. Although adherence to the GFD contributes to improving quality of life (QoL), in the long term, the restrictions imposed for treatment and surveillance can lead to psychological-overload symptoms and disordered eating (DE), negatively impacting the QoL of individuals with CD [7,8,9].

Disordered eating (DE) includes preoccupying eating behaviours, such as a spectrum of problematic eating behaviours and distorted attitudes towards food, weight, shape, and appearance. DE includes dieting; skipping meals; fasting; restricting food intake; eliminating specific foods or food groups; binge eating; use of diuretics, laxatives, and weight loss medications; and use of compensatory behaviours (purging and excessive exercising) [10,11]. DE also includes clinically diagnosed eating disorders (EDs), which are complex disorders encompassing constant disturbances in eating behaviours and impairment in psychological issues, such as anorexia nervosa, bulimia nervosa, binge eating disorder, avoidant restrictive food intake disorder, and other specified feeding disorders [5,6]. Studies have shown that DE is common in people with gastrointestinal disorders. The prevalence is estimated to be 55% among individuals with this diagnosis [7,12]. In people with CD, for example, DE is significantly more common than in the healthy population. Other studies indicate a variable and high prevalence of maladaptive eating disorders in individuals with CD, ranging from 8.88% to 43.5% [8,9,12]. In this sense, evaluating eating attitudes and behaviours among people with CD appears crucial to reduce the risk of DE and improve their nutritional quality and quality of life, potentially guiding clinical strategies and public policies in this area.

Research on eating attitudes and behaviours in people with CD is recent and has focused on the use of instruments most designed for the general population. Among the main general instruments applied are the Binge Eating Staircases (BES) [13], the Eating Attitudes Test (EAT-26) [14], and the Eating Disorder Inventory (EDI-2) [11,15,16]. Excessive concerns about GFD or hypervigilance are warning signs of maladaptive eating habits for people with CD, leading to increased symptoms of depression and decreased QOL [11,17,18,19]. Furthermore, avoiding foods other than gluten can be a sign of inappropriate food behaviours, such as avoidant/restrictive food intake disorder (ARFID), among individuals with CD [17]. For this reason, the Coeliac Disease Food Attitudes and Behaviours (CD-FAB) was developed in the UK. The CD-FAB is a scale specifically designed to evaluate food attitudes and behaviours among people with CD [20]. In the study using CD-FAB applied in the US, with 50 CD patients, higher scores were associated with decreased QOL, especially in the first year after diagnosis, and greater psychological suffering, such as anxiety, depression, and stress [20,21]. The CD-FAB is the only instrument specifically designed for adults with CD, demonstrating reliable and valid measures of food attitudes and behaviours, as well as evaluating concerns about food in individuals with CD. Initially developed in English, CD-FAB was previously translated and validated into Turkish; however, no versions are available in other languages or used in countries beyond the UK, the USA, and Turkey [20,21,22,23,24].

Given the importance of applying the same instrument across populations for comparison and the limited studies on this topic in Latin America, including the Brazilian CD population, this study aimed to translate and test the reliability of the CD-FAB in the Brazilian population. In Brazil, the estimated prevalence is similar to the global average, affecting about 2 million Brazilians, although most remain underdiagnosed [25]. Therapeutic success is also intrinsically linked to GFD adherence, which, in the Brazilian context, is permeated by socioeconomic and cultural challenges, such as limited knowledge about GFD, restricted access to and high cost of products, labelling, and the risk of cross-contamination, frequently resulting in low GFD adherence [26,27,28]. Given this, the accurate assessment of food attitudes and behaviours is fundamental for monitoring and clinical intervention, but the absence of psychometrically validated and culturally adapted instruments for Brazilian Portuguese represents a methodological gap. Therefore, the translation and cross-cultural adaptation of a questionnaire on food attitudes and behaviours is imperative to ensure the validity and reliability of research and clinical interventions targeting the Brazilian celiac population. The use of a specific tool to assess attitudes and behaviours regarding food benefits for individuals with CD, and research in the area, may guide public policies for this population.

## 2. Materials and Methods

### 2.1. Study Design and Ethical Approval

This cross-sectional study was performed in accordance with the Declaration of Helsinki and approved by the Research Ethics Committees of the Faculty of Health Sciences of the University of Brasília, CEP/FS (no. 7.806.366—8 May 2025). Volunteers received a consent form and signed it before participation. To ensure the validity of the translation and cultural adaptation of the CD-FAB into Brazilian Portuguese, the methodological process consisted of three main stages: translation and cultural adaptation of the CD-FAB, pretest to assess item clarity and comprehension, and assessment of the psychometric properties of the Brazilian version of CD-FAB.

### 2.2. Translation and Cultural Adaptation of the CD-FAB

The CD-FAB is an 11-item scale designed for people with CD aged 18 to 69, assessing food attitudes and behaviours resulting from beliefs about avoiding gluten contact and food safety with Likert-type options varying from 1 (strongly disagree) to 7 (strongly agree). The score is obtained using a Likert scale of seven points (1 “strongly disagree” to 7 “strongly agree”). The score ranges from 11 to 77 points [21], with high scores suggesting more maladaptive eating attitudes and behaviours. Clinical-relevance cut-offs have not yet been established. The translation and adaptation of the questionnaire followed the recommendation of Beaton et al. (2000) [29], with adaptation. The adaptation occurred in the pretesting stage, in which Beaton (2000) [29] recommends 30 participants, and we performed this step into two phases: (i) first (described in Section 2.3), we evaluated the understanding of the meaning of each item, following the NEEDs Centre protocol [30]; and second, (ii) the test–retest was performed with 27 participants (described in Section 2.4) [31].

In the translation process, two bilingual translators (Brazilian Portuguese/English), acting independently and without prior knowledge of the instrument, translated the CD-FAB, including the filling instructions and the items. Translator 1 was female, 24 *y*/*o*, Brazilian residing in the US, and had completed higher education. Translator 2 was male, 31 *y*/*o*, with a higher-education degree in English language/translation. Both followed the guidance to maintain one language that is accessible, close to the popular language, suitable to the target audience, and culturally adapted. Then, two other researchers analysed both versions, translated them, and, by consensus, developed a unified version. This version was then forwarded to a bilingual native translator of the original language, with no prior contact with the instrument, and who did not participate in the previous stages necessary for the back-translation. After that, two researchers compared the back-translated version (in English) with the original questionnaire and analysed the Brazilian-Portuguese translation version to make adjustments in case of non-conformities. The bilingual translators agreed on the final version as the last step in the translation process.

### 2.3. Pretest

After the instrument translation, a pretest was conducted with a convenience sample. At least five Brazilian CD individuals aged >18 and <69 years old, residing in Brazil, were invited to participate [30]. Due to the translation process of an instrument for individuals diagnosed with coeliac disease (which limits the sample), the pretest must be conducted with at least four native speakers who are unfamiliar with the instrument. Recruitment for this stage was conducted among celiac patients registered with the Brazilian celiac association (ACELBRA) via e-mail, ACELBRA’s social media, and messaging groups. Participants evaluate their understanding of the meaning of each item. Based on the participants’ responses, the researchers assessed the comments and adjusted the items, maintaining their meaning. The final translated version of each item in the translation table was recorded and then discussed it among researchers before the final approval of the translation [32,33,34].

The volunteers completed an online form containing a consent form and sociodemographic questions (age, sex, and income) to characterize participants, and then their understanding of each CD-FAB item was assessed using a Likert scale (0—I didn’t understand at all; 1—I understood very little; 2—I understood partially; 3—I understood almost everything; 4—I understood well; and 5—I understood completely). A field was left open for suggestions at the end of the questionnaire. When a participant provided scores between 0 and 3, the researchers discussed the item again, making suggested adjustments to improve clarity. Afterwards, everyone was asked to re-evaluate only the adjusted item. Items that received scores of 4 or 5 from at least 80% of the volunteers were considered clear and well understood [35].

### 2.4. Psychometric Evaluation of the Brazilian Version of CD-FAB

Psychometric evaluation was assessed through reproducibility and internal consistency, using responses from a test–retest with 27 new participants diagnosed with CD who met the eligibility criteria. Eligibility criteria at this stage were Brazilian CD individuals aged >18 and <69 years, residing in Brazil, who were unfamiliar with the instrument. In the instrument evaluation process, it is essential to evaluate reproducibility (reliability) in a small sample of participants to determine the correlation between two measurements made on the same subject at different times. This pilot reliability testing is required before performing the external validation of the instrument with a representative sample [36].

For this purpose, recruitment was conducted by sharing an online link and inviting interested parties to register. This was accompanied by media outreach through social and search channels, allowing direct contact with an association of people with CD. Eligible volunteers received an initial access link to the Brazilian version of CD-FAB (CD-FAB-BR). Without prior notice, they received a new link with the same instrument 48 h after the first fulfilment. They were asked to answer the questionnaire between 2 and 15 days. They collected information on sociodemographic characteristics (age, sex, and income) to characterize participants, and the CD-FAB instrument was applied.

### 2.5. Data Analysis

Descriptive data were presented in the form of frequencies and percentages (%) for categorical variables and means and standard deviations (SDs) for quantitative variables. The intraclass correlation coefficient (ICC) was used to assess the instrument’s reproducibility through a bidirectional mixed-effects model. ICC values between 0.75 and 0.9 indicate good test–retest reliability, while values above 0.90 indicate excellent reliability [37]. Cronbach’s alpha coefficient (α) was used to assess the internal consistency of the instrument, with values considered suitable greater than 0.7 [38]. The statistical analyses were carried out using the IBM Statistical Package for Social Sciences software version 25.0 (SPSS Inc., Chicago, IL, USA).

## 3. Results

**Figure 1:**
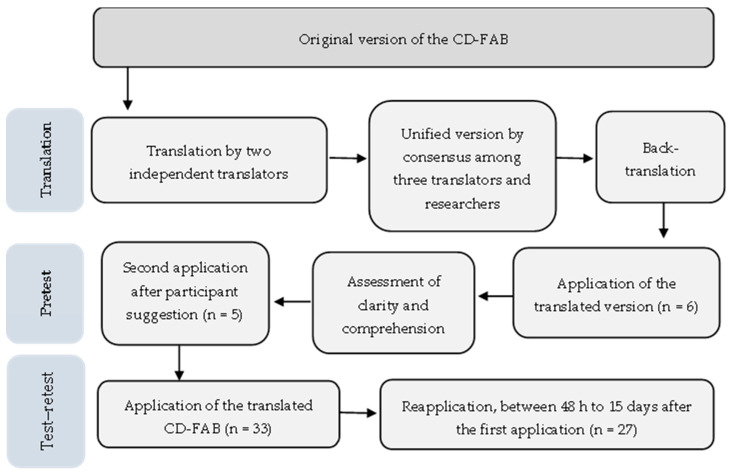
Flowchart for translation and validation of the CD-FAB.

A summary of the steps involved in the translation, cultural adaptation, and the reproducibility assessment of the CD-FAB, along with the number of participants at each step, is presented in Figure 1. The characterisation of participants in the pretest and test–retest is described in Supplementary Materials Tables S1 and S2, respectively.

The CD-FAB translation process confirms the attention paid to adapting the instrument to the Brazilian context. Table 1 shows that during translation, some expressions were discussed among translators to ensure cultural adaptation without altering meaning or rendering them hard to understand for the target audience.

### 3.1. Pretest

Among the 11 items of the CD-FAB, 9 showed good clarity and understanding, with 100% of participants marking 4 or 5 on the scale (5 “I understand totally” or 4 “I understand well”). Two items were modified because one participant (16.7%) marked 2 on the scale, indicating that the items were not comprehensible. The two items were as follows: “Mesmo já tendo me contaminado com glúten, não deixei de aproveitar a ida a restaurantes” (Being contaminated by gluten in the past has not stopped me from enjoying restaurants); and “ Se eu pergunto diretamente aos funcionários do local, geralmente consigo encontrar alimentos sem glúten para comer” (If I ask questions, I can normally find gluten-free food to eat). Therefore, the two items were reformulated by the group of experts and reapplied to the same sample of participants. Only five of the six previous participants agreed to participate in this phase. The characterisation of the pretest participants is presented in the Supplementary Materials (Table S1).

### 3.2. Reproducibility and Internal Consistency of the CD-FAB-BR

Thirty-three people completed the first questionnaire, of whom thirty-one were eligible to respond again within 15 days. However, only twenty-seven completed the second application (retest phase) and were included in the final analysis of reproducibility and internal consistency. The final sample (n = 27) is small and highly homogeneous (85% women with high educational level—Table S2). The mean age of the participants was 39.8 ± 14.8 years. Eighty-five per cent had a bachelor’s degree and a family income between BRL 8000 and BRL 24,800 (48%, n= 13; Supplementary Materials Table S2). The test–retest reproducibility of the CD-FAB-BR indicates excellent agreement between responses, with an intraclass correlation coefficient (ICC) of 0.928 (95% CI: 0.842–0.967), exceeding 0.70 (Table 2). Furthermore, good internal consistency was achieved with a Cronbach’s alpha of 0.807 (95% CI: 0.677–0.899).

## 4. Discussion

This study validated the first and only instrument assessing coeliacs’ food attitudes and behaviours designed explicitly for Brazilian adults with CD. The translation demonstrated clarity and a good understanding, and was it culturally adapted for the Brazilian population. With the version in Brazilian Portuguese, it was possible to maintain the structure of the items, adjusting them as needed to ensure clarity and understanding for the target audience. Despite the existence of tools to assess DE, they are limited in adequately capturing the specificities in CD patients [29]. The pretest results ensured the cultural and semantic pertinence of the CD-FAB-BR, demonstrating that the volunteers adequately understood the instrument [29]. Cultural adaptation is a fundamental step in the translation and validation of instruments [39]. The main challenge in translating and culturally adapting the instrument was finding words that conveyed the same meaning without altering the core issue and were understandable to the Brazilian population. In particular, the item “If I ask questions, I can normally find gluten-free food to eat” was unclear about whether it referred to restaurants, which sparked debate among the researchers. Furthermore, using accessible language for diverse audiences was a concern for the translators.

The test–retest results demonstrated excellent reliability, high reproducibility, and internal consistency of the adapted instrument (ICC = 0.928 and Cronbach’s alpha = 0.807) [37,38]. The reliability was close to that of the original instrument (Cronbach’s alpha = 0.89) [20]. A similar result was found in the CD-FAB validation study conducted in Turkey, which reported an ICC of 0.811 and Cronbach’s alpha of 0.842 [22]. Reliability evaluates an instrument’s capacity to produce consistent results when applied under similar conditions. It is considered an essential phase prior to larger studies and can be conducted with a small sample [40]. This study evaluated reliability using the test–retest method and assessing reproducibility and internal consistency. Reproducibility evaluates the stability of participants’ responses over time and must be evaluated over an interval of 2 to 14 days between the two applications [41].

The mean interval in the test–retest study was 6.07 ± 4.51 days, following the recommendations for analysing response stability [41,42]. The ICC of 0.928 indicates excellent stability of responses over time [42]. To account for potential memory bias and minor modifications to health during the response interval, which may influence the results, we also calculated internal consistency using Cronbach’s alpha. Internal consistency refers to the degree of correlation among the items of an instrument [42,43]. Cronbach’s alpha of 0.807 indicates a strong correlation among the items of CD-FAB-BR [42,43]. These findings indicate that CD-FAB-BR has excellent psychometric properties, with a high ICC and a high Cronbach’s alpha, which consolidate its reliability for assessing food attitudes and behaviours in coeliac adult patients. Good psychometric properties enable the use of CD-FAB-BR in clinical practice, which may enhance GFD monitoring and expand comprehensive care for individuals with CD. The CD-FAB-BR might help health professionals in the advanced identification of DE, including emotional distress, food avoidance, and hypervigilance.

The use of CD-FAB across different languages and CD populations allows for the identification of atypical eating attitudes and behaviours resulting from surveillance around beliefs about gluten contamination and food safety that are not detected by existing measures of eating disorders [20,21], justifying its importance in the Brazilian CD context, since hypervigilance, feeding avoidance, and psychological suffering can affect people who must follow a strict GFD [17,18,19]. The use of instruments not specific to CD cannot identify risks associated with different disorders or classify them incorrectly as disorderly behaviours inherent to CD management [18,44]. In this way, the validation of the instrument enables the evaluation of behaviours that directly affect QoL and psychological well-being in people with CD.

In our study, most of the sample (85%) was composed of females, similar to other studies with individuals with CD [19,45,46,47,48]. It is probably due to their concerns about health and the higher prevalence of CD and ED [2,11,12]. CD is more prevalent among females [2], who also show a higher prevalence of anorexic and bulimic attitudes, as well as binge eating [10,11,12,49]. Factors related to dietary management, low adherence to the gluten-free diet, and gastrointestinal symptoms most influenced the increase in scores for anorexia and bulimia nervosa. Furthermore, psychological distress was strongly associated with binge eating, which may be linked to stress associated with a chronic disease controlled by diet [8,9,49]. It is known that the prevalence and risks of eating disorders in people with chronic diseases are higher than in the general population, according to a meta-analysis [9]. The management and handling of the gluten-free diet by people with coeliac disease have a significant negative impact that extends beyond the diet itself, requiring a more comprehensive clinical approach, including an emphasis on the burden of the imposed dietary restriction, and constant monitoring of the diet [17,24].

The adherence to a GFD remains the primary challenge in treating CD, primarily due to factors such as the need for constant caution with food security, reading food labels, questioning the presence of gluten in eating out, and concerns about cross-contact. This continuous surveillance can lead to psychological suffering and predispose individuals to DE [11,18,19].

### Study Limitations

This study has some limitations. The test–retest sample was predominantly female and consisted of a small sample size. In addition, the retest occurred 48 h to 15 days after the initial test (mean interval of 6.07 ± 4.51 days). While this falls within commonly recommended windows for test–retest reliability, the minimum interval of only 48 h may be too short to avoid memory effects that could artificially inflate reliability estimates. Another limitation relates to the use of a convenience sample. While it allows for easy tracking of participants and ensures they receive an invitation to a second response promptly, this approach may have limited sample diversity and resulted in a small and homogeneous sample. The sample of 27 participants in the test–retest analysis is limited for drawing strong conclusions about the reliability of the CD-FAB-BR, but it is acceptable for reliability pilot testing before future larger studies for validation. Despite this, 27 duplicated answers are considered sufficient for this step [50]. Furthermore, the predominant social class among most participants may mask the behaviour of people with lower socioeconomic status and lower levels of education. Additionally, we were unable to contact the instrument’s author to contribute to the back-translation, despite several attempts via e-mail. This research focused on psychometric analyses of the reproducibility and internal consistency of the CD-FAB, which require small samples and do not include analyses related to variables, instruments, or measures. The nature of a cross-sectional study limits the ability to draw conclusions about changes in food attitudes and behaviours in CD patients over time. Future longitudinal studies should be conducted to evaluate changes in psychological aspects and gluten-free dietary management in adults with CD.

## 5. Conclusions

This study was successful in translating, culturally adapting, and conducting a pilot reliability testing of the CD-FAB for the Brazilian population with CD (CD-FAB-BR), thereby introducing the first specific instrument to assess food attitudes and behaviours of adults with CD in Brazil. The psychometric evaluation results showed excellent reliability of the CD-FAB-BR. The use of CD-FAB-BR may yield data that help develop more effective interventions for adults with coeliac disease, guide health professionals in advising CD patients on GFD, and prompt governments to formulate public policies for this population. In addition to contributing to optimal disease management, it supports research in the area. However, it is still necessary to expand future research on the behaviour and attitudes of people with CD in Brazil, particularly by increasing the number of participants and applying the instrument to a representative sample of Brazilian coeliac patients for external validation and cultural and regional sensitivity analyses.

## Figures and Tables

**Table 1:** CD-FAB translation process from the original version to the Brazilian-Portuguese version.

Response Options and Items	English Original	Translation 1	Translation 2	Translation 1–2	English Back-Translation	Revised Translation After Pretest
Directions	Instructions: This questionnaire is designed to explore food attitudes and beliefs in coeliac disease.	Instruções: Este questionário foi pensado pra explorar atitudes alimentares e crenças na doença celíaca.	Instruções: este questionário é projetado para explorar atitudes e crenças alimentares relacionadas à doença celíaca.	Este questionário foi desenvolvido para explorar atitudes e crenças alimentares relacionadas à doença celíaca	This questionnaire was developed to explore dietary attitudes and beliefs related to coeliac disease.	Este questionário foi desenvolvido para explorar atitudes e crenças alimentares relacionadas à doença celíaca.
	Some questions may not apply to you; this is because we are trying to assess a range of beliefs about coeliac disease and managing the gluten-free diet.	Algumas perguntas podem não se aplicar a você; isso ocorre porque estamos tentando avaliar uma série de crenças sobre a doença celíaca e o manejo da dieta sem glúten.	Algumas perguntas podem não se aplicar a você; isso ocorre porque estamos tentando avaliar uma variedade de crenças sobre a doença celíaca e sobre como administrar a dieta livre de glúten.	Algumas perguntas podem não se aplicar a você; isso ocorre porque estamos tentando avaliar uma série de crenças sobre a doença celíaca e como gerenciar a dieta sem glúten.	Some questions may not apply to you; this happens because we are trying to evaluate a range of beliefs about coeliac disease and how to manage a gluten-free diet.	Algumas perguntas podem não se aplicar a você; isso ocorre porque estamos tentando avaliar uma série de crenças sobre a doença celíaca e como gerenciar a dieta sem glúten.
	Please fill out the form below as accurately, honestly, and completely as possible.	Por favor, preencha o formulário abaixo da forma mais precisa, honesta e completa possível.	Por favor, preencha o formulário abaixo da forma mais precisa, honesta e completa possível.	Por favor, preencha o formulário abaixo da forma mais precisa, honesta e completa possível.	Please fill out the form below as accurately, truthfully, and completely as possible.	Por favor, preencha o formulário abaixo da forma mais precisa, honesta e completa possível.
	There are no right or wrong answers.	Não existem respostas certas ou erradas.	Não há respostas certas ou erradas.	Não existem respostas certas ou erradas.	There are no right or wrong answers.	Não existem respostas certas ou erradas.
	All of your responses are confidential.	Todas as suas respostas são confidenciais.	Todas as tuas respostas são confidenciais.	Todas as suas respostas são confidenciais.	All your responses will be kept confidential by the researchers.	Todas as suas respostas são confidenciais.
	Please tick the box that best describes your response to the question.	Por favor, assinale a caixa que melhor descreve a sua resposta à questão.	Por favor, marque a caixa que melhor descreve sua resposta para a questão.	Por favor, marque a caixa que melhor descreve sua resposta para a questão.	Please mark the option that best describes your answer to each question.	Por favor, marque a caixa que melhor descreve sua resposta para a questão.
Response options	Strongly agree (7)	Concordo muito (7)	Concordo muito (7)	Concordo muito (7)	strongly agree (7)	Concordo totalmente (7)
	Agree (6)	Concordo (6)	Concordo (6)	Concordo (6)	Agree (6)	Concordo (6)
	Somewhat agree (5)	Concordo um pouco (5)	Concordo de alguma forma (5)	Concordo de alguma forma (5)	Slightly agree (5)	Concordo de alguma forma (5)
	Neither agree nor disagree (4)	Não concordo e nem discordo (4)	Nem concordo nem discordom (4)	Nem concordo nem discordo (4)	Neither agree nor disagree (4)	Nem concordo nem discordo (4)
	Somewhat disagree (3)	Discordo um pouco (3)	Discordo de alguma forma (3)	Discordo de alguma forma (3)	Slightly disagree (3)	Discordo de alguma forma (3)
	Disagree (2)	Discordo (2)	Discordo (2)	Discordo (2)	Disagree (2)	Discordo (2)
	Strongly disagree (1)	Discordo muito (1)	Discordo muito (1)	Discordo muito (1)	Strongly disagree (1)	Discordo totalmente (1)
Items	***1**—Because of my coeliac* *disease…*	*1—Em razão da minha* *doença celíaca…*	*1—Devido à minha doença* *celíaca…*	*1—Devido a minha doença* *celíaca…*	1—Due to coeliac disease…	Devido à doença celíaca…
	I get concerned being near others when they are eating gluten.	Eu fico preocupado estando perto de outras pessoas quando elas estão comendo glúten	Eu fico preocupado de estar perto de outras pessoas quando elas estão comendo glúten.	Eu fico preocupado de estar perto de outras pessoas quando elas estão comendo glúten.	I worry about being around other people when they are eating food that contains gluten.	eu fico preocupado de estar perto de outras pessoas quando elas estão comendo glúten.
	I am afraid to eat outside my home.	Eu tenho medo de comer fora da minha casa.	Eu tenho medo de comer fora de casa.	Eu tenho medo de comer fora de casa.	I am afraid of eating out.	eu tenho medo de comer fora de casa.
	I am afraid to touch gluten-containing foods.	Eu tenho medo de encostar em comidas que contêm gluten.	Eu tenho medo de entrar em contato com comidas que contêm glúten.	Eu tenho medo de entrar em contato com comidas que contêm glúten.	I am afraid of having contact with food that contains gluten.	eu tenho medo de entrar em contato com comidas que contêm glúten.
	I get worried when eating with strangers.	Eu fico preocupado quando como algo com pessoas estranhas.	Eu fico preocupado quando estou comendo com estranhos.	Eu fico preocupado quando como com estranhos.	I get worried when I am eating with strangers.	eu fico preocupado quando como com estranhos.
	I find it hard to eat gluten-free foods that look like the gluten-containing foods that made me ill in the past.	Acho difícil comer alimentos sem glúten que se pareçam com os alimentos que contêm glúten que me deixaram doente no passado.	Eu tenho dificuldade de comer comidas sem glúten que se parecem com as comidas com glúten que me adoeceram no passado.	Acho difícil comer alimentos sem glúten que parecem com os alimentos que contêm glúten que me deixaram doente no passado.	I found it is difficult to eat gluten-free foods that are similar to foods that contain gluten that have made me sick in the past.	acho difícil comer alimentos sem glúten que parecem com os alimentos que contêm glúten que me deixaram doente no passado.
	I will only eat food that I have prepared myself.	Eu só vou comer comida que eu mesmo preparei.	Eu só como comida que eu mesmo preparei.	Eu só como comida que eu mesmo preparei.	I only eat food prepared by myself.	Só como comida que eu mesmo preparo.
	My concerns about cross-contamination prevent me from going to social events involving food.	Minhas preocupações com a contaminação cruzada me impedem de ir a eventos sociais que envolvam alimentos.	Minhas preocupações sobre contaminação cruzada me impedem de ir a eventos sociais que envolvem comida.	Minhas preocupações com a contaminação cruzada me impedem de ir a eventos sociais que envolvam alimentos.	My concern with cross-contamination from gluten is attending social events that serve food.	Minhas preocupações com a contaminação cruzada me impedem de ir a eventos sociais que envolvam alimentos.
	*2—Despite having coeliac* *disease…*	*2—Apesar de ter doença* *celíaca…*	*2—Apesar de ter doença* *celíaca…*	*2—Apesar de ter doença* *celíaca…*	Despite having coeliac disease…	Apesar de ter doença celíaca…
	I enjoy going out for meals as much as I did before my diagnosis *.	Eu gosto de sair pra comer tanto quanto gostava antes de ser diagnosticada(o).	Eu gosto de sair para comer tanto quanto gostava de sair antes do meu diagnóstico.	Eu gosto de sair pra comer tanto quanto gostava antes de ser diagnosticada(o).	I like to go out to eat as much as I liked before the diagnosis.	Eu gosto de sair para comer tanto quanto gostava antes de ser diagnosticada(o).
	I am comfortable eating gluten-free food from other people’s kitchens *.	Sinto-me confortável comendo alimentos sem glúten da cozinha de outras pessoas *.	Eu fico confortável comendo comidas sem glúten da cozinha de outras pessoas *.	Sinto-me confortável comendo alimentos sem glúten da cozinha de outras pessoas *.	I feel comfortable eating gluten-free food prepared by other people.	Sinto-me confortável comendo alimentos sem glúten da cozinha de outras pessoas.
	Being contaminated by gluten in the past has not stopped me from enjoying restaurants *.	Estar contaminado por glúten no passado não me impediu de desfrutar de restaurantes *.	Ser contaminado(a) pelo glúten no passado não me impediu de apreciar restaurantes.	Ser contaminado(a) pelo glúten no passado não me impediu de apreciar restaurantes.	In the past, gluten contamination has not stopped me from enjoying eating in restaurants.	Mesmo já tendo me contaminado com glúten, não deixei de aproveitar a ida a restaurantes
	If I ask questions, I can normally find gluten-free food to eat *.	Se eu fizer perguntas, normalmente consigo encontrar alimentos sem glúten para comer *.	Se eu perguntar, eu posso encontrar normalmente comida sem glúten para comer.	Se eu fizer perguntas, normalmente consigo encontrar alimentos sem glúten para comer *.	If I ask around, I can usually find gluten-free food.	Se eu pergunto diretamente aos funcionários do local, geralmente consigo encontrar alimentos sem glúten para comer.
	Reverse items with * and add all scores to make total score.	Inverta os itens com * e some todas as pontuações para obter a pontuação total.	Inverta os itens com * e some todas as pontuações para obter a pontuação total.	Inverta os itens com * e some todas as pontuações para obter a pontuação total.	Invert items with * and add all the scores to get the total score.	Inverta os itens com * e some todas as pontuações para obter a pontuação total.

**Table 2:** Reproducibility of the CD-FAB-BR.

	CD-FAB-BR (n = 27)
Tests means (SD ^1^)	45.07 (12.03)
Retest means (SD)	45.03 (13.57)
ICC ^2^ (95% CI)	0.928 (0.842–0.967)

^1^ Standard deviation; ^2^ intraclass correlation coefficient (ICC).

## Data Availability

The original contributions presented in this study are included in the article/Supplementary Materials. Further inquiries can be directed to the corresponding authors.

## Supplementary Materials

The following supporting information can be downloaded at https://www.mdpi.com/article/10.3390/nu18010162/s1. Table S1: Characterization of participants in the pre-test. Table S2: Characteristics of the participants included in the reproducibility and internal consistency assessment stage of the CD-FAB-BR.
