# Supplementary material for: Cultural Adaptation and Reliability Testing of the Coeliac Disease Food Attitudes and Behaviours Scale in Brazil
Source: Nutrients. 2026 Jan 3;18(1):162. doi: 10.3390/nu18010162 (PMC12787950; doi:10.3390/nu18010162)
Supplement: Supplementary file 1 [file nutrients-18-00162-s001.zip › nutrients-3988505-supplementary.pdf]

Supplementary file.

Table S1: Characterization of participants in the pre-test.

		n = 6
Age		26.8 (± 7)
Birth sex	Male	4 (66.7%)
Educational level	High School	1 (16.7%)
	Undergraduate	5 (83.3%)
Household income per month[1]	Up to BRL 3,300	2 (33.3%)
	BRL 3,300 to BRL 8,000	3 (50%)
	BRL 8,000 to BRL 24,800	1 (16.7%)
	More than BRL 24,800	0
	Prefer not to inform	0

[1]: Brazilian Real is the official currency of Brazil, and USD 1.00 = BRL 5.39 (15 October 2025)

Table S2: Characteristics of the participants included in the reproducibility and internal consistency assessment stage of the CD-FAB-BR.

		n = 27
Age		39.8 (± 14.8)
Birth sex	Female	23 (85.2%)
Educational level	High School	1 (3.7%)
	Undergraduate	3 (11.1%)
	Graduate	23 (85.2%)
Household income per month[1]	Up to BRL 3,300	4 (14.8%)
	BRL 3,300 to BRL 8,000	6 (22.2%)
	BRL 8,000 to BRL 24,800	13 (48.1%)
	More than BRL 24,800	3 (11.1%)
	Prefer not to inform	1 (3.7%)

[1]: Brazilian Real is the official currency of Brazil, and USD 1.00 = BRL 5.39 (15 October 2025).
